# Non-linear association between CD4+ T-cell counts and mortality risk in people living with HIV: evidence from a 10-year cohort study

**DOI:** 10.3389/fmed.2025.1502804

**Published:** 2025-02-14

**Authors:** Ruohan Sun, Huangchao Jia, Qiujia Kang, Yanmin Ma, Zichen He, Xiuxia Ma, Jun Yuan, Huijun Guo, Qianlei Xu, Yantao Jin

**Affiliations:** ^1^The First Affiliated Hospital of Henan University of CM, Zhengzhou, China; ^2^Henan University of Chinese Medicine, Zhengzhou, China; ^3^Henan Center for Disease Control and Prevention, Zhengzhou, China

**Keywords:** non-linear associations, CD4+ T-cell count, mortality risk, Cox proportional hazards regression model, restricted cubic spline

## Abstract

**Background:**

The study aimed to investigate the dose–response association between CD4+ T-cell counts and mortality risk in people living with HIV (PLHIV).

**Methods:**

Data on PLHIV who had a high prevalence of acquired immunodeficiency syndrome (AIDS) were retrospectively collected from the routine treatment database in Henan Province, China, covering the period from October 2003 to October 2021. We randomly selected 1,000 PLHIV from age groups 30, 40, 50, and 60 years who met the inclusion criteria as study participants. The Kaplan–Meier analysis, the Cox proportional hazards regression model, and the restricted cubic spline (RCS) model were employed to analyze the association between CD4+ T-cell counts and mortality risk in PLHIV.

**Results:**

A total of 4,000 participants were enrolled in the study, with a follow-up period of 28,158 person-years. During this period, there were 941 (23.5%) deaths, resulting in a mortality rate of 3.34 per 100 person-years. The mean duration of follow-up was 6.77 ± 3.45 years, and the cumulative survival rate was 76.5%. The Cox proportional hazards regression model revealed that as the CD4+ T-cell count increased, the hazard ratio (HR) decreased. The results of the RCS model demonstrated a non-linear association between CD4+ T-cell counts and mortality risk in PLHIV, with cutoff values for each age group being 382, 332, 334, and 215 cells/μL. The non-linear curve indicated that the HR decreased as the CD4+ T-cell counts increased. However, once the CD4+ T-cell counts reached their respective cutoff values, the curve showing the reduction in mortality risk began to flatten.

**Conclusion:**

There was a non-linear association between CD4+ T-cell counts and mortality risk in PLHIV. Although the cutoff values vary across age groups, they consistently remain close to 350 cells/μL. Therefore, considering a threshold of CD4+ T-cell count <350 cells/μL across various age groups is crucial as a strategic approach to mitigate mortality rates among PLHIV.

## Background

Acquired immunodeficiency syndrome (AIDS) is an infectious disease caused by human immunodeficiency virus (HIV). According to the World Health Organization, there were approximately 39.9 million (36.1 million–44.6 million) cases of people living with HIV (PLHIV) by the end of 2023, with 1.3 million (1 million-1.7 million) new infections and 630,000 (500,000-820,000) deaths (1). AIDS remains a significant global public health challenge.

With the widespread use of CD4+ T-cell count detection technology, the CD4+ T-cell count has become a crucial indicator for assessing the immune status and clinical stage of HIV-infected individuals (2), initiating antiretroviral therapy (ART) and evaluating the therapeutic effect (3). Numerous studies have investigated the association between CD4+ T-cell counts and mortality risk among PLHIV. However, many of these studies treated the CD4+ T-cell count as a categorical variable, often overlooking its dynamic fluctuations and their evolving impact on mortality risk (4, 5). This dynamic trajectory may exhibit non-linear characteristics, which are often overlooked in linear analyses. The restricted cubic spline (RCS) model effectively integrates spline functions into generalized linear models (e.g., logistic regression and Cox proportional hazards regression) to elucidate the dose–response association between independent and dependent variables (6).

In this study, a 10-year retrospective cohort design was based on the routine treatment database, with a specific focus on PLHIV aged 30, 40, 50, and 60 years to control for age effects. The RCS model was used to analyze the dose–response association between CD4+ T-cell counts and mortality risk in PLHIV.

## Methods

### Research design

According to a previous study, the 10-year mortality rate among PLHIV and related diseases was 32.1% (7). Consequently, the sample size was determined using the following formula:


$$N=\frac{Z_{\alpha }^{2}\;p\left(1-p\right)}{d^{2}}$$

where *α* = 0.05, *d* = 0.1p, and *p* = 0.321. This calculation yielded a sample size of 813 cases; however, considering a 15% loss to follow-up, at least 957 individuals were required for this study. The study focused on PLHIV from a specific area in Henan and was conducted from October 2003 to October 2021. We randomly selected 1,000 PLHIV from the age groups of 30, 40, 50, and 60 years who met the inclusion criteria and were followed up for 10 years, until October 2021, or until death. Age served as the grouping factor: “30 years,” “40 years,” “50 years,” and “60 years.”

First-line ART regimens before 2008 consisted of [azidothymidine (AZT) or stavudine (D4T)] + [didanosine (DDI) or lamivudine (3TC)] + [nevirapine (NVP) or efavirenz (EFV)]. The second edition of the *National Free ART Guideline*, released in 2008, was revised to include [oftenofovir (TDF) or azidothymidine (AZT)] + lamivudine (3Tc) + [efavirenz (EFV) or nevirapine (NVP)] (8).

### Study population

PLHIV in Henan Province, China, which has a high prevalence of AIDS, were included in the study if they tested positive for HIV using Western blotting and were aged 30, 40, 50, or 60 years. Participants were required to have a baseline CD4+ T-cell count at the start of the study. Individuals with follow-up periods of less than 1 month or incomplete information on variables were excluded from the study.

### Data collection

Information regarding the study individuals was extracted from the routine treatment database named “AIDS Prevention and Control Information System.” This information included birth date, sex, marital status, occupation, educational level, transmission, HIV-positive confirmation time, CD4+ T-cell count, detection time, ART initiation time, treatment withdrawal time, death time, and cause of death.

### Data analysis

The latest CD4+ T-cell count recorded within 6 months before or after cohort commencement was used as the CD4+ T-cell value for the study analysis, which was divided into evenly spaced intervals of 100 cells/μL for interpretation. Continuous variables were described as means ± standard deviations, while categorical variables were described as frequencies and percentages. The life table method was used to calculate mortality density, and the Kaplan–Meier method was used to construct survival curves. The Cox proportional hazards regression model was used to analyze factors influencing mortality in PLHIV, utilizing hazard ratios (HRs) and corresponding 95% confidence intervals (CIs) to describe the association. The RCS model was used to analyze the dose–response association between the CD4+ T-cell counts and mortality risk in PLHIV across the different age groups, with density diagrams depicting the distribution of the CD4+ T-cell count for each group. Generally, R^2^ and Dxy were used to evaluate the RCS model. R^2^ indicates the goodness of fit, representing the proportion of variation explained by the model, while Dxy represents the model’s discrimination ability. Larger values indicate a better model. The model with the highest R^2^ and Dxy values was considered the optimal model after the RCS analysis. The data were analyzed using R4.2.0, and a *p*-value of <0.05 was considered statistically significant.

## Results

### Summary of the study population

In each age group, 1,000 participants were included, for a total of 4,000 participants. Of these, 941 (23.5%) deaths were recorded, with a total of 28,158 person-years accrued during the follow-up, resulting in a mortality rate of 3.34/100 person-years. The mean duration of the follow-up was 6.77 ± 3.45 years. Among the cohort, 2,218 (55.5%) were male participants, 1,782 (44.5%) were female participants, and 3,559 (89.0%) were farmers. Within the study group, 2,316 (57.9%) participants were infected through blood transmission, 3,497 (87.4%) participants had been HIV-positive for more than 8 years, and 2,515 (62.9%) participants had received ART for more than 8 years. The mean ART duration for the participants aged 30, 40, 50, and 60 years was 7.2, 20.3, 28.7, and 32.5 months, respectively. The mean HIV-positive duration for these age groups was 30.6, 44.4, 44.8, and 50.8 months, respectively. The mean CD4+ T-cell count was 374 ± 227 cells/μL. The distribution of PLHIV based on their baseline CD4+ T-cell counts is as follows:

0–100 cells/μL: 369 individuals (9.2%),

101–200 cells/μL: 554 individuals (13.9%),

201–300 cells/μL: 742 individuals (18.6%),

301–400 cells/μL: 740 individuals (18.5%),

401–500 cells/μL: 605 individuals (15.1%),

501–600 cells/μL: 403 individuals (10.1%), and

>600 cells/μL: 587 individuals (14.7%). Detailed information on the baseline characteristics and mortality rates in the subgroups of PLHIV is shown in Table 1.

**Table 1:** Detailed information on the baseline characteristics and mortality rates in the subgroups of PLHIV.

Variables	Total *N* = 4,000	Death *N* = 941	Follow-up person-year	Mortality rate (per 100 person-years)
Age group				
30 years	1,000 (25.0%)	137 (14.6%)	6,689	2.05
40 years	1,000 (25.0%)	193 (20.5%)	8,028	2.40
50 years	1,000 (25.0%)	248 (26.4%)	7,008	3.54
60 years	1,000 (25.0%)	363 (38.6%)	6,433	5.64
CD4+ T-cell count (cells/μL)				
0–100	369 (9.2%)	183 (19.4%)	2,035	8.99
101–200	554 (13.9%)	190 (20.2%)	3,796	5.01
201–300	742 (18.6%)	178 (18.8%)	5,417	3.29
301–400	740 (18.5%)	149 (15.8%)	5,493	2.71
401–500	605 (15.1%)	110 (11.7%)	4,349	2.53
501–600	403 (10.1%)	57 (6.1%)	2,905	1.96
>600	587 (14.7%)	74 (7.9%)	4,162	1.78
Sex				
Male	2,218 (55.5%)	572 (60.8%)	14,294	4.00
Female	1,782 (44.5%)	369 (39.2%)	13,864	2.66
Marital status				
Married	2,608 (65.2%)	614 (68.1%)	18,986	3.23
Single/widow	1,392 (34.8%)	327 (31.9%)	9,171	3.57
Occupation				
Farmer	3,559 (89.0%)	909 (96.5%)	25,880	3.51
Others	441 (11.0%)	32 (3.5%)	2,277	1.41
Educational level				
≤6 years	2,071 (51.8%)	604 (64.2%)	15,224	3.97
>6 years	1,929 (48.2%)	337 (35.8%)	12,934	2.61
Transmission				
Blood	2,316 (57.9%)	693 (73.6%)	18,360	3.77
Others	1,684 (42.1%)	248 (26.4%)	9,797	2.53
HIV-positive confirmation time (year)				
0–3	239 (6.0%)	62 (6.6%)	1,443	4.30
>3 ~ 8	264 (6.6%)	68 (7.2%)	1,689	4.03
>8	3,497 (87.4%)	811 (86.2%)	25,026	3.24
Duration of antiretroviral therapy (year)				
0	1,034 (25.9%)	272 (28.9%)	7,601	3.58
>0 ~ 3	207 (5.2%)	55 (5.8%)	1,261	4.36
>3 ~ 8	244 (6.1%)	52 (5.5%)	1,664	3.12
>8	2,515 (62.9%)	562 (59.7%)	17,632	3.19

### Cumulative survival rates of PLHIV across different age groups and CD4+ T-cell count categories

**Figure 1:**
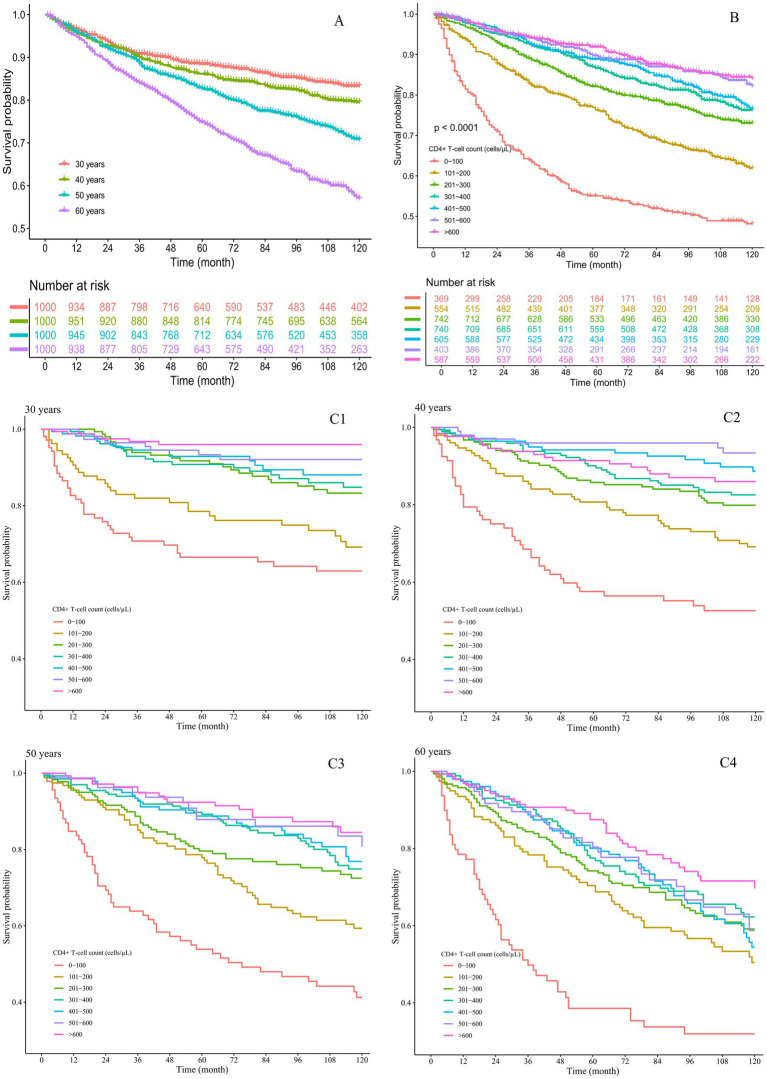
Cumulative survival rates of the PLHIV. **(A)** By different age groups; **(B)** by different CD4+ T-cell count groups; **(C)** by both age and CD4+ T-cell count groups, with subgroups **(C1)** 30 years, **(C2)** 40 years, **(C3)** 50 years, and **(C4)** 60 years.

Among the 4,000 participants, the cumulative survival rate was 76.5%. The cumulative survival rates for PLHIV aged 30, 40, 50, and 60 years were 83.3, 79.5, 70.7, and 57.0%, respectively. PLHIV aged 60 years demonstrated lower survival rates compared to other age groups (Figure 1A). The cumulative survival rates for PLHIV with baseline CD4+ T-cell counts of 0–100, 101–200, 201–300, 301–400, 401–500, 501–600, and > 600 cells/μL were 48.2, 61.9, 73.1, 76.2, 76.7, 82.1, and 84.1%, respectively, indicating that a higher baseline CD4+ T-cell count was associated with an improved survival rate (Figure 1B). As the follow-up time increased, the survival curves for the CD4+ T-cell count groups across all four age groups showed a downward trend (Figures 1C1–C4). Among these, the 60-year-old group with a CD4+ T-cell count of 0–100 cells/μL exhibited the fastest decline in the survival curve (Figure 1C4).

### Factors influencing the survival rates of PLHIV

The univariable Cox proportional hazards model showed that PLHIV aged 50 and 60 years had a higher mortality risk compared to those aged 30 and 40 years. A CD4+ T-cell count >100 cells/μL, being a woman, working in occupations other than farming, having more than 6 years of education, transmission through routes other than blood, and time on ART >8 years decreased the risk of mortality.

**Figure 2:**
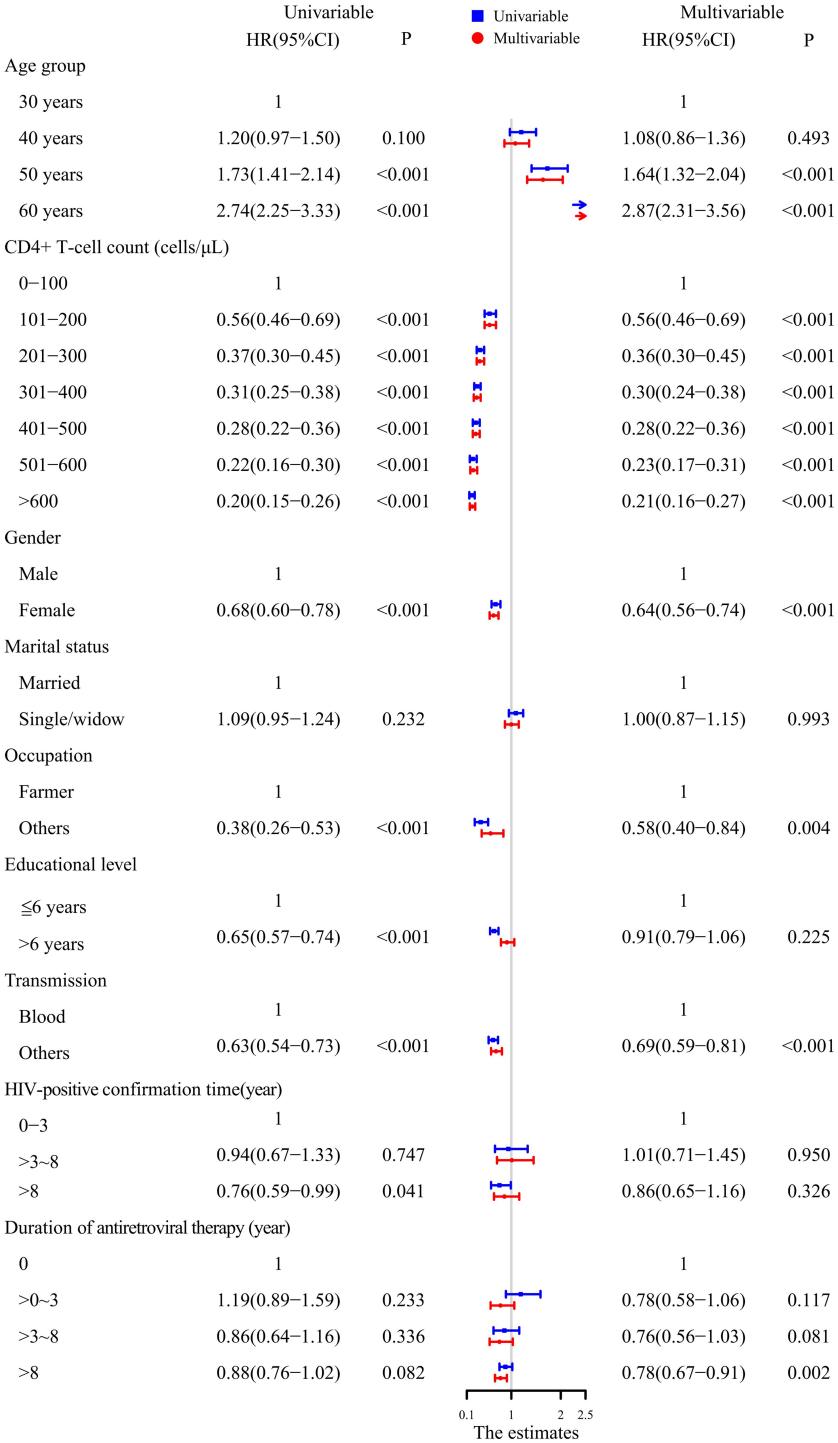
Analysis of the factors influencing survival in PLHIV using the Cox proportional hazards model.

After adjusting for factors such as sex, marital status, occupation, education level, transmission, HIV-positive confirmation time and time on ART, the multivariable Cox proportional hazards regression model showed that PLHIV aged 50 and 60 years, a CD4+ T-cell count >100 cells/μL, being female, working in occupations other than farming, transmission through routes other than blood, and time on ART >8 years were all independently associated with mortality. In addition, as the CD4+ T-cell count increased, the HR value decreased. The results are shown in Figure 2.

### Non-linear association between CD4+ T-cell counts and mortality risk in PLHIV

**Figure 3:**
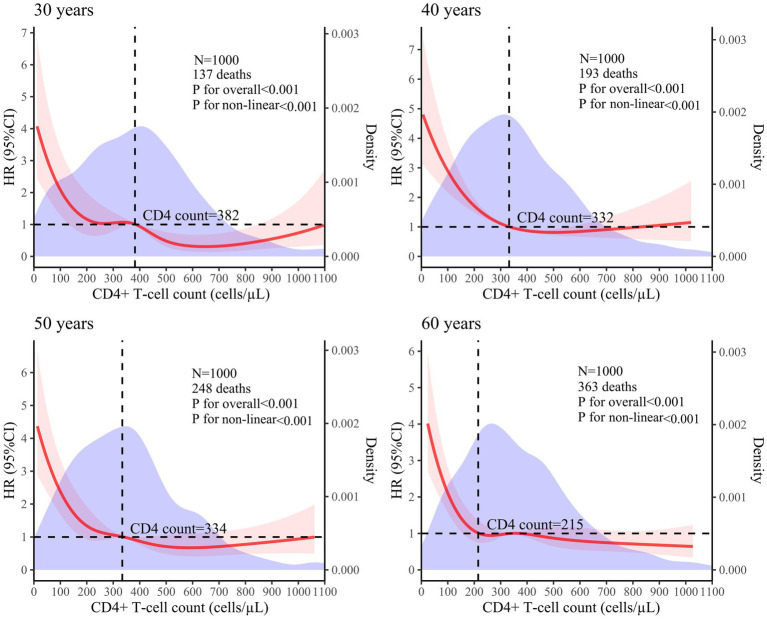
Analysis of the non-linear association between CD4+ T-cell counts and mortality risk in PLHIV based on the RCS model (The red solid line represents the HR value, the red shaded area corresponds to its 95% CI, and the purple area represents the density of different CD4+ T-cell counts).

A 5-knot RCS model was used for the 30-year, 50-year, and 60-year age groups. A 3-knot RCS model was used for the 40-year age group (results detailed in Table 2). The optimal RCS model revealed a non-linear association between the CD4+ T-cell counts and mortality risk in the PLHIV across the different age groups, with cutoff values of 382, 332, 334, and 215 cells/μL for the 30-, 40-, 50-, and 60-year age groups, respectively. The non-linear curve showed that the HR decreased as the CD4+ T-cell count increased. However, once the CD4+ T-cell counts reached their respective cutoff values, the curve indicating the mortality risk reduction began to flatten, as shown in Figure 3.

**Table 2:** Evaluation of RCS models with different knots across the four age groups.

Group	3 knots		4 knots		5 knots	
	*R* ^2^	Dxy	*R* ^2^	Dxy	*R* ^2^	Dxy
30 years	0.456	0.449	0.458	0.456	0.478	0.480
40 years	0.404	0.424	0.403	0.424	0.403	0.423
50 years	0.343	0.399	0.343	0.402	0.344	0.402
60 years	0.183	0.277	0.198	0.278	0.202	0.279

## Discussion

The Cox proportional hazards regression model indicated that as the CD4+ T-cell count increased, the HR value decreased; however, the HR value did not exhibit a proportional reduction, suggesting a non-linear association between the CD4+ T-cell counts and mortality risk in PLHIV. This finding aligns with those of previous research (9, 10). While regression models are frequently utilized to explore associations between CD4+ T-cell counts and mortality risk, these models often presuppose a linear association (11). If actual data do not conform to this assumption, simply converting the CD4+ T-cell count into a categorical variable may not only overlook or discard the continuous nature of the data but also introduce new biases, thereby potentially affecting the accuracy and reliability of the analysis results. In contrast, the RCS model can treat CD4+ T-cell counts as continuous variables to effectively model their non-linear association with mortality risk through a smooth curve.

Previous studies have demonstrated that age is a significant risk factor for mortality in PLHIV (12, 13). These studies typically categorized PLHIV by age groups, such as Yue Tingting’s study, which divided PLHIV into four groups: under 30 years, 30–39 years, 40–49 years, and 50 years and older (14). Tian Bo used 50 years as the cutoff point, dividing age groups into two: below 50 years and above 50 years (15). Jun-fan Pu categorized age into four groups: 15–29 years, 30–44 years, 45–59 years, and 60 years and older (16). This approach often employs broad intervals of 10 or 15 years, and in some cases, a singular boundary at 50 years, potentially exacerbating disparities in the target analysis variables across different age cohorts (17). Therefore, this study adopted a more refined division strategy, focusing specifically on ages 30, 40, 50, and 60 years to control for potential confounding effects of age on the research results.

The RCS model showed that once the CD4+ T-cell counts reached the respective cutoff values—382 cells/μL for 30 years, 332 cells/μL for 40 years, 334 cells/μL for 50 years, and 215 cells/μL for 60 years—the curve depicting the reduction in mortality risk tended to flatten. Research has indicated that as age increases, the likelihood of opportunistic infections and complications also rises, leading to higher mortality risks (18, 19). Therefore, it is expected that the cutoff value would decrease with advancing age.

Although the cutoff values for each age group vary, they consistently remain around 350 cells/μL. A CD4+ T-cell count of 350 cells/μL is a critical threshold in AIDS-related research. Several studies have used survival curves to clearly illustrate that PLHIV with baseline CD4+ T-cell counts ≥350 cells/μL have higher survival rates throughout the observation period compared to those with counts <350 cells/μL. In addition, the Cox proportional hazards model revealed that the PLHIV with a CD4+ T-cell count <350 cells/μL exhibited a significantly higher mortality risk than those with a count ≥350 cells/μL (16, 20). A study aimed at identifying the determinants of tuberculosis co-infection among PLHIV in Papua found that HIV-infected individuals with a baseline CD4+ T-cell count <350 cells/μL were at a significantly higher risk of opportunistic infections compared to those with a CD4+ T-cell count ≥350 cells/μL (21). Some studies on complications associated with HIV infection have suggested that a CD4+ T-cell count <350 cells/μL can accelerate hepatitis progression (22) and is a risk factor for malignancies (23) and cervical cytological abnormalities (24) in PLHIV. Studies on the impact of HIV-1 genetic diversity on disease progression (25) and the efficacy and safety of a simplified lamivudine plus dolutegravir dual therapy in HIV-1-Infected patients (26) have also identified that a CD4+ T-cell count <350 cells/μL is a risk factor that impedes CD4 + T lymphocyte recovery. Research on pregnancy in women has found that a lower pregnancy incidence (27) and adverse pregnancy outcomes (28) are associated with a CD4+ T-cell count <350 cells/μL. Therefore, considering these findings along with the cutoff value, it is widely recognized that a CD4+ T-cell count <350 cells/μL signifies an elevated risk of mortality and necessitates disease prevention strategies.

In summary, there was a non-linear association between CD4+ T-cell counts and mortality risk in PLHIV. As CD4+ T-cell counts fall below their respective cutoff values, the mortality risk increases significantly. Notably, despite variations in cutoff values across age groups, they consistently remain around 350 cells/μL. Consequently, it is imperative to concentrate on populations whose mortality risk increases rapidly. Comprehensively considering cutoff values—specifically targeting a CD4+ T-cell count <350 cells/μL—is vital for reducing the mortality rate of PLHIV. There are certain limitations to this study. Firstly, it was a retrospective study based on real-world data, which might have resulted in incomplete data collection and insufficient exclusion of confounding factors, such as reasons for attrition and comorbidities. Secondly, only participants aged 30, 40, 50, and 60 years were included, and age was treated as a discrete variable—for example, a person aged 30 years and 11 months was placed in the 30-year age group although they were closer to 40 years old— potentially limiting the generalizability of the results. Thirdly, the single data source might have limited the variability regarding the infection routes. In addition, the study spanned a considerable period but did not account for the progress of the ART protocols over time, which might have introduced bias in mortality risk. Considering these limitations, it is necessary to conduct prospective, multicenter cohort studies to analyze the association between CD4+ T-cell counts and mortality risk of PLHIV, thereby addressing the shortcomings of this study.

## Data Availability

The original contributions presented in the study are included in the article/supplementary material, further inquiries can be directed to the corresponding authors.
